# Effect of far ultraviolet light emitted from an optical diffuser on methicillin-resistant *Staphylococcus aureus in vitro*

**DOI:** 10.1371/journal.pone.0202275

**Published:** 2018-08-10

**Authors:** David Welch, Manuela Buonanno, Igor Shuryak, Gerhard Randers-Pehrson, Henry M. Spotnitz, David J. Brenner

**Affiliations:** 1 Center for Radiological Research, Columbia University Irving Medical Center, New York, New York, United States of America; 2 Department of Surgery, Columbia University Irving Medical Center, New York, New York, United States of America; Universidade Federal do Rio de Janeiro, BRAZIL

## Abstract

Drug-resistant bacteria such as methicillin-resistant *Staphylococcus aureus* (MRSA) are a target for new antimicrobial technologies. Far-UVC technology is an emerging disinfection method that directly kills microorganisms using light. In contrast with conventional UV sterilization, far-UVC light has antimicrobial capabilities without apparent harm to mammalian cells. This study examines the application of 224 nm far-UVC light delivered from a laser using an optical diffuser towards the goal of protecting against bacterial invasion around skin penetrating devices. Delivery of far-UVC using a laser and optical fibers enables exposure to unique geometries that would otherwise be shielded when using a lamp. Testing of the bactericidal potential of diffusing the far-UVC laser output over a large area was tested and yielded qualitative area killing results. The killing of MRSA using this method was also examined using an *in vitro* survival assay. Results followed a classic log-linear disinfection model with a rate constant of k = 0.51 cm^2^/mJ, which corresponds to an inactivation cross section of D_90_ = 4.5 mJ/cm^2^. This study establishes far-UVC delivered from a laser through an optical diffuser as a viable solution for disinfection of susceptible regions such as around catheters, drivelines, or other skin penetrating medical devices.

## Introduction

Infections from drug-resistant bacteria such as methicillin-resistant *Staphylococcus aureus* (MRSA) present major health care challenges with over 23,000 cases resulting in death in the United States each year [1]. Fighting these infections is a strain on limited healthcare resources considering the average length of a hospital stay is estimated at more than 11 days longer for infected individuals than those without infection [2]. Substantial financial burdens are also a consequence, with estimated associated costs in the US approaching $10 billion annually [3]. While significant resources have been applied to combat infection from drug-resistant bacteria, and incremental gains have been reported, effective resolutions remain elusive.

One method of microbial disinfection is using ultraviolet (UV) light, conventionally using low-pressure mercury lamps emitting primarily at 254 nm. UV light at 254 nm can efficiently kill both drug-sensitive and multi-drug-resistant bacteria [4], as well differing strains of viruses [5]. However, the widespread use of germicidal ultraviolet light has been very limited because conventional UVC light sources are a human health hazard, being both carcinogenic and quite damaging to eye tissue [6, 7].

By contrast, it has been shown that far-UVC light generated by filtered excimer lamps emitting in the 207 to 222 nm wavelength range, efficiently kills drug-resistant bacteria, without apparent harm to exposed mammalian skin [8–11]. The biophysical reason is that, due to its strong absorbance in biological materials, far-UVC light does not have sufficient range to penetrate through even the outer layer (stratum corneum) on the surface of human skin, nor the outer tear layer on the outer surface of the eye, neither of which contain living cells; however, because bacteria and viruses are typically of micron or smaller dimensions, far-UVC light can still efficiently traverse and inactivate them [8–10]. Furthermore, recent work has shown that irradiation of an open wound, which did not have a protective layer of stratum corneum, with 222 nm light did not induce DNA damage in targeted cells while still showing bactericidal control [11].

Adventitious infections due to pathogens such as MRSA are especially prominent within wounds surrounding catheters, drivelines for left ventricular assist devices, and lines for hemodialysis [12–14]. In these cases, a far-UVC light source such as an excimer lamp would not be sufficient for disinfection because the implanted lines would act as shielding for much of the wound. Additionally, the far-UVC light cannot penetrate the skin to disinfect within the wound. One possible solution to these situations is to direct light around and within complex geometries using fiber optics. The fiber optics, which can be coupled with a far-UVC laser as a source of light, could be manipulated to deliver light to multiple locations around an area prone to infection. Pairing the fiber optic with a diffusing element capable of emitting the light radially from the fiber would allow for application over a large target area within the percutaneous site that would be impossible to expose otherwise.

This study examines the use of far-UVC light generated from a laser source to be delivered via fiber optics to an optical diffuser element for bactericidal applications. These studies are performed *in vitro* as a first step to demonstrate the efficacy of this method. The development of this new technique to reduce infection at skin entry sites while also being potentially safe for human exposure is significant for the fight against drug-resistant bacteria, particularly in hospital settings.

## Materials and methods

### Far-UVC source and diffuser

The far-UVC laser module (He-Ag 70-224SL, Photon Systems, Covina, CA) used in this study emits at 224 nm and operates in a pulsed output mode. The laser was set to run with a buss voltage of 420 V, 15 A of current, a pulse width of 100 μs, and a pulse frequency of 20 Hz. A laser to multimode fiber coupler (HPUC-25-225-M-10BQ-2, Oz Optics, Ottawa, Ontario, Canada) with an f = 10 mm biconvex lens reduced the 3 mm output beam diameter into a 400 μm/440 μm/480 μm (core/cladding/coating) solarization resistant fiber with a diffuser on the distal end (Molex, Phoenix, AZ). The fiber has a numerical aperture of 0.22 and a length of approximately 1 meter. The cylindrical diffuser is made of fused silica which has been inscribed with micro-grooves using a laser and measures 1 mm in diameter and 5 cm long.

### Far-UVC dosimetry

Measurement of far-UVC exposure was performed using our previously published methods utilizing UVC sensitive film [15, 16]. This method was chosen because commercially available UV power meters, which usually integrate power over a single photodiode, do not measure the spatial power distribution with sufficient accuracy to characterize the unique power output of the optical diffuser. The film from Ashland Specialty Ingredients (Bridgewater, NJ) is referred to as unlaminated Gafchromic EBT3 (Product Code 849952). This specialty film is essentially one half of the regular Gafchromic EBT3 film; it is simply a 14 μm thick active layer on top of a 125 μm polyester substrate. The active layer contains a proprietary mixture of active material, marker dye, stabilizers, and other components. In our previous work, we reported on the utility of this film for UV exposure measurement [15]. The film showed a response to exposures from wavelengths ranging from 207 nm to 328 nm and exhibited a dynamic range extending up to as high as 100 mJ/cm^2^. The film is extremely sensitive, with radiant exposures in the range of μJ/cm^2^ detectable for 222 nm light. Our previous work also showed that the film has a wavelength dependent response thus it is important to calibrate with a source emitting a spectrum that closely matches the analyzed light source. The film has a high spatial resolution with the ability to resolve features to at least 25 μm. The polyester layer is blocking to UV radiation so the active region must be oriented towards the source for accurate measurement. When the film is radiated on the side with the active region it exhibits a nearly ideal cosine response like other radiochromic films [17, 18].

Calibration of the UV sensitive film was performed through a series of exposures using an excimer lamp (High Current Electronics Institute, Tomsk, Russia) with a krypton-chlorine (Kr-Cl) gas mixture emitting principally at 222 nm. A custom bandpass filter (224NB7, Omega Optical, Brattleboro, VT), CWL of $222_{-1}^{+2}$ nm and FWHM of $7_{-1}^{+2}$ nm, was used with the Kr-Cl lamp to isolate the 222 nm peak [19]. The film response between the calibration wavelength of 222 nm and the measured wavelength of 224 nm from the laser is not significantly different thus this calibration is appropriate [15]. Optical power measurements for the calibration were performed using an 818-UV/DB low-power UV enhanced silicon photodetector with an 843-R optical power meter (Newport, Irvine, CA). A range of radiant exposures, from 3.6 μJ/cm2 up to 281.6 mJ/cm2, were performed on the film to define a response curve. Films were scanned as 48 bit RGB TIFF images at 150 dpi using an Epson Perfection V700 Photo flatbed scanner (Epson, Suwa, NGN, Japan) and analyzed with radiochromic film analysis software (radiochromic.com) [20] to calculate the total exposure based on measured changes in optical density.

### MRSA preparation

We used methicillin-resistant *S*. *aur*eus (MRSA USA300, multilocus sequence type 8, clonal complex 8, staphylococcal cassette chromosome mec type IV), for its clinical relevance [21–23]. Fresh colonies of MRSA were inoculated into tryptic soy broth (TSB) (Thermo Scientific, Pittsburgh, PA, USA) and grown overnight at 37° C. The culture was then resuspended in fresh broth and grown to mid-log phase for 2 hours. Bacteria were collected by centrifugation, washed, resuspended in broth, and adjusted to an optical density at 600 nm of 0.5 which corresponds to 10^6^ colony formation units (CFU) per ml of Hanks' balanced salt solution (HBSS).

### Area disinfection assay

**Fig 1:**
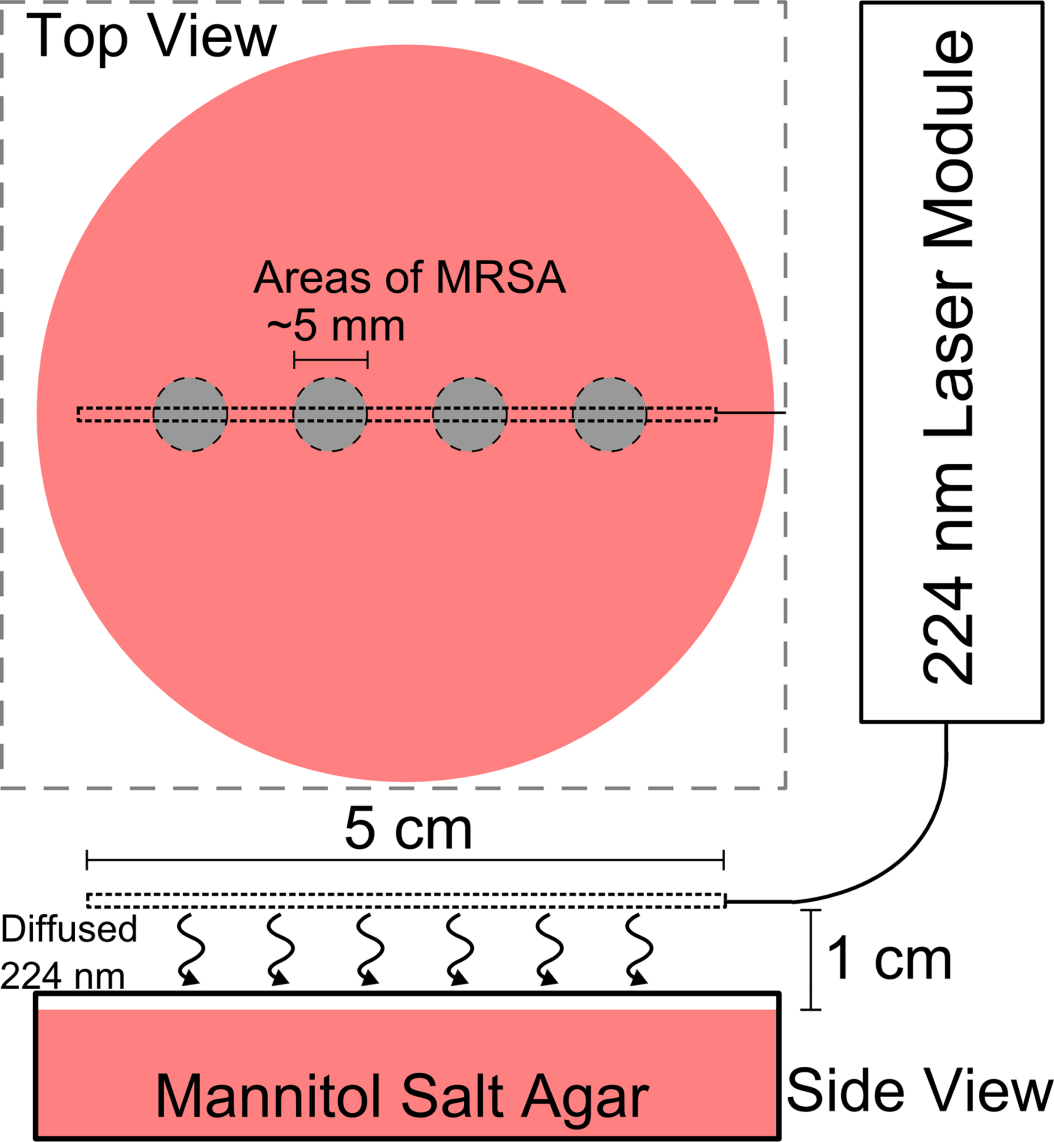
Experimental setup for MRSA area disinfection assay and survival assay. The experimental setup for the MRSA survival assay includes a far-UVC laser module, a fiber optic cable connecting the laser to an optical diffuser, and an agar filled petri dish. The diffuser was suspended in air 1 cm above the surface of the agar in the dish. For the cell survival assay the diffuser was aligned on top of four identical 5-μl aliquots of 10^6^ CFU/ml of MRSA, each with an area of about 5 mm in diameter. An identical equipment setup was used for the area disinfection assay except 50 μl of MRSA was spread to cover the entire surface of the dish.

Assessment of the ability to diffuse bactericidal far-UVC over an area was performed using the equipment setup shown in Fig 1. Standard 60-mm diameter plates completely filled beforehand with mannitol salt agar (BD Diagnostic System, Sparks, MD, USA) served as the disinfection surface. A diffuser was suspended in air 1 cm away from the agar surface. One 50-μl aliquot of 10^6^ CFU/ml of MRSA was spread evenly across the entire agar surface. The laser was powered for 2 hours to expose the sample and then the dish was incubated overnight to allow bacteria growth. A second dish was handled in parallel but not exposed to the laser as a control of the experimental conditions.

### Survival assay

Five 5-μl aliquots of 10^6^ CFU/ml of MRSA were dispensed onto a single agar plates as shown in Fig 1. Each aliquot covered a circular area approximately 5 mm in diameter. Four aliquots were aligned directly under the length of the diffuser to allow simultaneous exposures while a fifth aliquot was placed in an unexposed area and used as positive control (i.e. zero UV dose). All aliquots of bacterial solution were allowed to dry for 30 min and then exposed to the far-UVC light (or unirradiated in the case of the zero dose). Variation of the far-UVC dose was obtained by exposing each aliquot for 0.5, 1, 1.5, or 2 h which corresponded to an average far-UVC dose over the aliquot area of 4.1, 8.1, 12.2, or 16.3 mJ/cm^2^, respectively. At the end of each sampling time, a swab of a specific aliquot was taken and resuspended in TSB; 50 μl of serial dilutions were then spread onto tryptic soy agar plates containing 5% sheep blood (BD Diagnostic System, Sparks, MD, USA) using a Drigalski spatula. Two plates were used for each exposure condition and were incubated overnight at 37° C, with colony counting performed the following day.

### Data analysis

Bacterial survival fraction was expressed as the proportional reduction in CFU/cm^2^, relative to unexposed controls for a given experiment. Values represent the average ± SEM of the independent experiments. Survival values (*S*) were calculated for each repeat experiment and natural log transformed (ln(*S*)) to bring the error distribution closer to normal [24]. Linear regression was performed using these normalized ln(*S*) values as the dependent variable and UV dose (*D*, mJ/cm^2^) as the independent variable. Microorganism susceptibility, i.e. survival in UV-C light, traditionally follow first-order kinetics and thus the bacterial survival was fitted to the equation

ln(S)=−k×D, [Eq 1]

with *k* the UV rate constant or susceptibility factor (cm^2^/mJ) and *D* the dose (mJ/cm^2^) [25]. The regression was performed with the intercept term set to zero, which assumes 100% relative survival at zero UV dose. Bootstrap 95% confidence intervals for the parameter *k* were calculated using R 3.2.3 software [26]. MRSA inactivation cross-section D_90_, or the UV dose that kills 90% of the bacteria, was calculated as D_90_ = − ln (1 − 0.9) / k [25].

## Results

### Area disinfection assay

**Fig 2:**
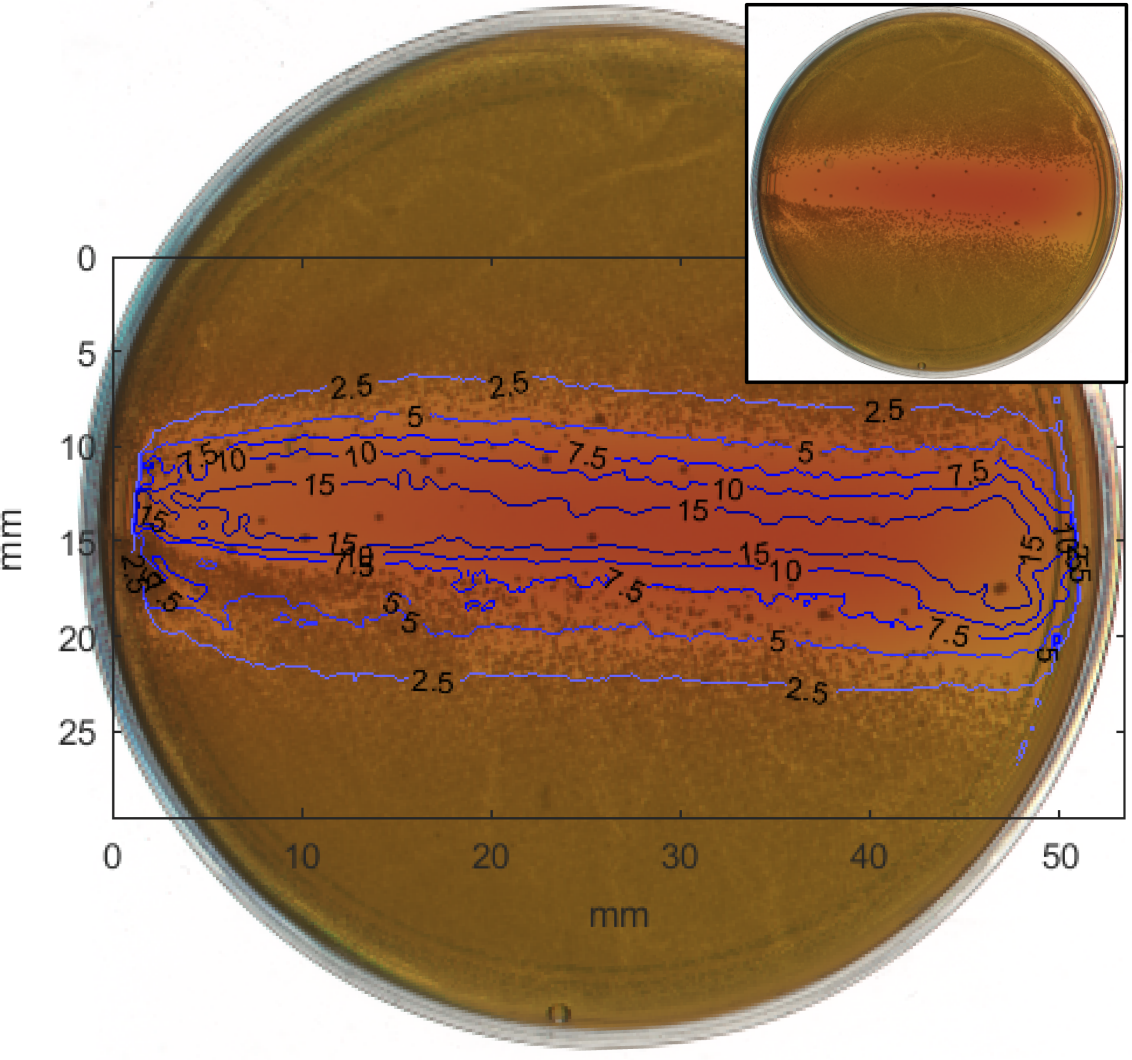
Area of MRSA sterilization after diffused far-UVC exposure. A picture of the MRSA growth pattern after exposure to diffused far-UVC light shows the ability to sterilize a large area using a single laser source. The MRSA appears as darker regions against the lighter colored agar. A contour map of the exposure dose, calculated using film exposures with the same experimental setup and expressed in mJ/cm^2^, is overlaid to show that variations in dose have a clear correlation with the killing efficiency. A picture of the dish without doses overlaid is shown in the top right.

The killing of MRSA across a surface is shown in Fig 2. An unmodified picture of the bacteria plate is shown in the inset of the figure. Dark areas on the figure indicate areas of bacterial growth. The figure includes an overlay of contour lines which indicate the levels of far-UVC exposure in mJ/cm^2^ for the two-hour test as calculated using film dosimetry. The area of highest exposure coincides with the area directly beneath the cylindrical diffuser. Less far-UVC exposure to an area on the surface corresponds to an increase in the amount of MRSA growth. Irregularities in the exposure pattern are due to physical variations in the diffuser itself that impacted the uniformity of the diffusion of light.

### Survival assay

**Fig 3:**
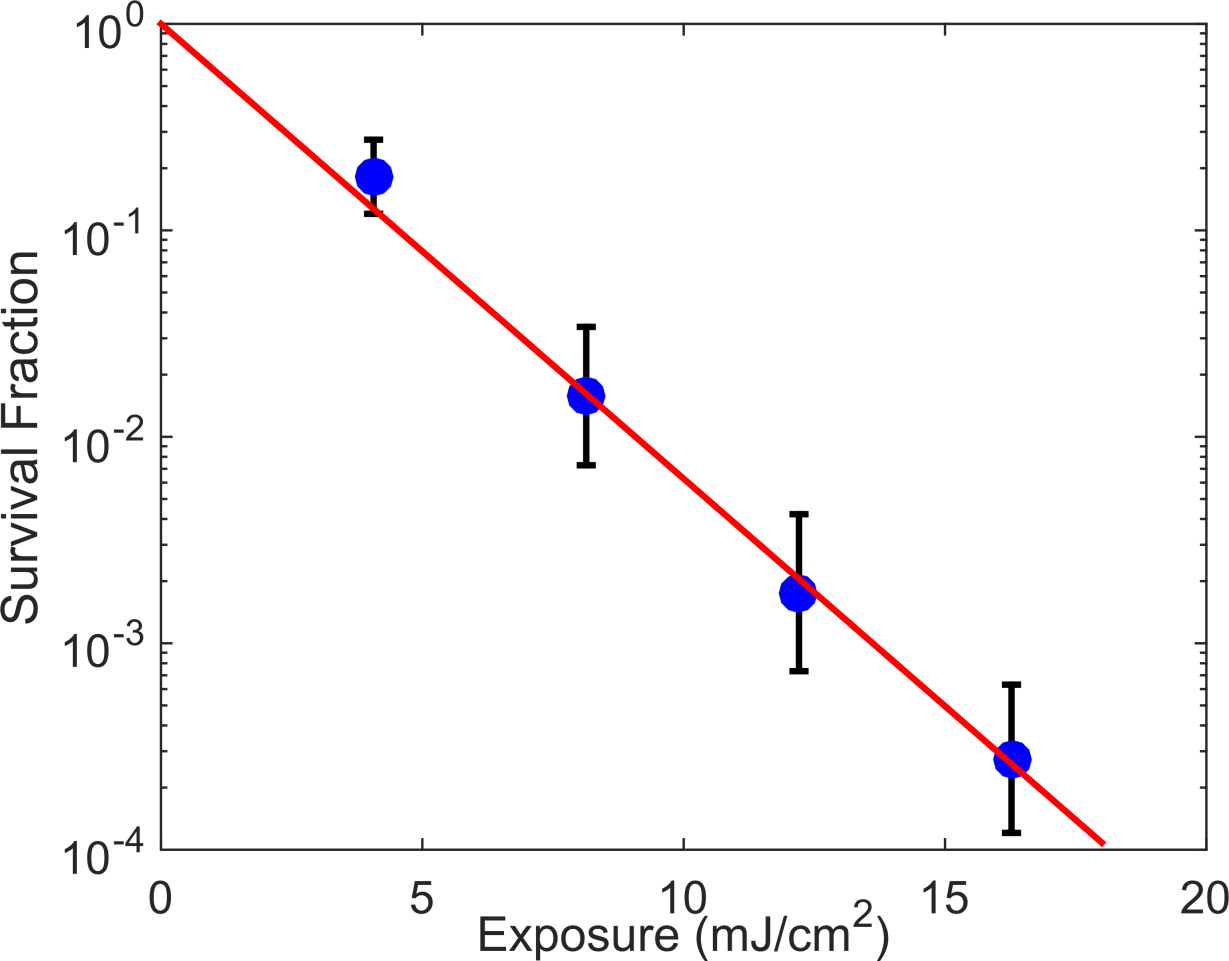
MRSA survival fraction for far-UVC exposure doses. Results for the survival assay for the killing of MRSA on a surface using far-UVC light emitted from a diffuser are plotted as bacterial survival fraction relative to zero-dose controls for the different exposures. The means and standard errors refer to triplicate repeat studies and the line represents the best-fit regression to Eq 1. The data follow a log-linear decay with a rate constant of 0.51 cm^2^/mJ. The calculated exposure dose required to kill 90% of bacteria is D_90_ = 4.5 mJ/cm^2^.

Fig 3 shows MRSA killing as a function of exposure to far-UVC light generated by a 224-nm laser and delivered through a diffuser. Raw data of the bacterial counts for each set of experiments and the corresponding log reduction is given in Table 1. The reduction of bacteria survival followed a classical log-linear UV disinfection model [25], with rate constant k = 0.51 cm^2^/mJ (standard error = 0.03, R^2^ = 0.76) with a 95% bootstrapped confidence interval of 0.44 cm^2^/mJ to 0.57 cm^2^/mJ. The rate constant corresponds to inactivation cross-sections of D_90_ = 4.5 mJ/cm^2^. A line plotting best-fit regression of the inactivation rate is included on Fig 3.

**Table 1:** Raw bacteria count data obtained from the survival assay experiments.

	Dose *(mJ/cm^2)*	Bacteria count *(CFU/ml)*	Log10 reduction from control
Experiment 1	0	380000	
	8.1	32000	1.07
	12.2	2800	2.13
	16.3	120	3.50
Experiment 2	0	750000	
	4.1	61000	1.09
	8.1	1500	2.70
	12.2	190	3.60
	16.3	20	4.57
Experiment 3	0	3600000	
	4.1	830000	0.64
	8.1	71000	1.71
	12.2	29200	2.09
	16.3	2020	3.25
Experiment 4	0	1030000	
	4.1	330000	0.49
	8.1	19100	1.73
	12.2	650	3.20
	16.3	1260	2.91

## Discussion

This study shows for the first time that far-UVC irradiation delivered using fiber optics is a promising technology in preventing infections caused by bacterial contamination. Delivery of a far-UVC light from a laser, through an optical fiber, and emitted from a diffuser is a unique application of this technology. Assembly of the experimental setup, including an optical fiber and diffuser capable of operating with far-UVC wavelengths, is the first demonstration of a general approach that could be adapted into various light delivery geometries. The area killing assay results shown in Fig 2 illustrate that this simple setup using only a single far-UVC laser source is capable of killing bacteria *in vitro* over an area much larger than the original laser beam diameter. The effectiveness of killing MRSA with far-UVC delivered in this manner was quantified through the survival assay. The disinfection rate constant of 0.51 cm^2^/mJ, with D_90_ = 4.5 mJ/cm^2^, is comparable to surface disinfection studies done on antibiotic-susceptible *S*. *aureus* with germicidal light at 254 nm emitted from a lamp, which had a rate constant k = 0.72 cm^2^/mJ with D_90_ = 3.2 mJ/cm^2^ [25]. The difference in performance of the two wavelengths for surface disinfection of MRSA is consistent with 254-nm light having a longer penetration distance than the 224-nm light and thus being more effective. Overall, the qualitative area assay results in Fig 2 displayed general agreement with the quantified survival response assay results.

Antisepsis using far-UVC light is an exciting option for use directly on and within tissue because, unlike germicidal UV at 254 nm, it does not produce biological damage to exposed mammalian cells and tissues. The technology is also unobtrusive, both because of the small size and the potential to not require additional protective equipment for the patient or caregiver, so it could be integrated into existing medical setups with minimal interference to existing protocols. Additionally, because far-UVC technology works via physical inactivation it is a potentially powerful tool against drug-resistant bacteria.

Delivery of far-UVC from a laser source using fiber optics is highly applicable to disinfecting difficult geometries. Optical components such as fibers and diffusers are small enough in size that they can be integrated around and within many of the pieces of medical equipment that are highly prone to infection. One example application is around a skin penetrating catheter which could be outfitted with diffusing elements surrounding the cylindrical tubing, thus providing the bactericidal effects directly at the interface between the skin and the device. Fiber optics provide the opportunity to use a single laser source to distribute light to numerous areas using established methods; creative applications of optics would allow for modifying this technique to fit most medical equipment already in use. These advances in delivery of far-UVC directly to an infection prone area are necessary because far-UVC wavelengths are unable to penetrate through most materials including various biological fluids. The lack of transmission of far-UVC through many biological materials, which is the basis for the indicated safety of this technology, therefore requires unobstructed delivery to target microbes for effective disinfection.

Recent advances in far-UVC laser technology have shown promise for shrinking both the size and the cost of the laser module to be better suited for widespread adoption. For example, Sharp Laboratories in Europe has produced compact UVC lasers that are about the size of a AA battery [27]; conversely, the laser module used in this work is 10 cm × 10 cm × 70 cm and 3.6 kg. Smaller laser modules could enable creating disinfection systems with far-UVC technology that are easily portable and thus able to be applied to a much wider range of applications.

Future work with far-UVC lasers for disinfection would benefit from improved light diffusing technology that is better suited for this application. Because of the difficulty in working with far-UVC wavelengths, largely due to solarization effects reducing transmittance [25], the optical components commercially available are limited. New diffusers that were more robust and more conformable to the unique biological environments that they will be used in would also advance this technology. Nevertheless, the potential of far-UVC as a safe, effective technology that assuages the desire for an antimicrobial tool that functions independent of acquired drug resistance is worth continued investigation.

## Conclusion

This study demonstrates that far-UVC light emitted from a laser and delivered using an optical diffuser is an effective tool to kill MRSA *in vitro*. The dose required to kill bacteria on a surface with far-UVC is slightly higher than with conventional UVC at 254 nm; however, given the lack of harmful biological effects with far-UVC, it is an attractive option for disinfection within wounds. The ability to diffuse the laser output over a large area makes this a viable solution for disinfection of infection prone tissues, such as around a catheter or other skin penetrating medical device.
